# Controversy on health-based guidance values for bisphenol A—the need of criteria for studies that serve as a basis for risk assessment

**DOI:** 10.1007/s00204-024-03778-3

**Published:** 2024-05-28

**Authors:** Marcel Leist, Andrea Buettner, Patrick Diel, Gerhard Eisenbrand, Bernd Epe, Petra Först, Tilman Grune, Dirk Haller, Volker Heinz, Michael Hellwig, Hans-Ulrich Humpf, Henry Jäger, Sabine E. Kulling, Angela Mally, Doris Marko, Ute Nöthlings, Elke Röhrdanz, Joachim Spranger, Stefan Vieths, Wim Wätjen, Jan G. Hengstler

**Affiliations:** 1 Division for In Vitro Toxicology and Biomedicine, Department of Biology, University of Konstanz, Universitaetsstrasse 10, 78464 Konstanz, Germany; 2 Chair of Aroma and Smell Research, Friedrich-Alexander-Universität Erlangen-Nürnberg, Henkestrasse 9, 91054 Erlangen, Germany; 3 Fraunhofer Institute for Process Engineering and Packaging IVV, Giggenhauser Strasse 35, 85354 Freising, Germany; 4 Department of Molecular and Cellular Sports Medicine, Institute of Cardiovascular Research and Sports Medicine, German Sport University Cologne, Am Sportpark Müngersdorf 6, 50933 Cologne, Germany; 5 Kühler Grund 48/1, 69126 Heidelberg, Germany; 6 Institute of Pharmaceutical and Biomedical Sciences, University of Mainz, Staudingerweg 5, 55128 Mainz, Germany; 7 Food Process Engineering, TUM School of Life Sciences, Technical University of Munich, Weihenstephaner Berg 1, 85354 Freising, Germany; 8 German Institute of Human Nutrition Potsdam-Rehbrücke (DIfE), Arthur-Scheunert-Allee 114-116, 14558 Nuthetal, Germany; 9 Chair of Nutrition and Immunology, Technical University of Munich, Gregor-Mendel-Strasse 2, Freising, Germany; 10 ZIEL Institute for Food and Health, Technical University of Munich, Weihenstephaner Berg 1, 85354 Freising, Germany; 11 DIL German Institute of Food Technology, Professor-von-Klitzing-Strasse 7, 49610 Quakenbrück, Germany; 12 Chair of Special Food Chemistry, Technical University Dresden, Bergstrasse 66, 01062 Dresden, Germany; 13 Institute of Food Chemistry, Universität Münster, Corrensstrasse 45, 48149 Münster, Germany; 14 University of Natural Resources and Life Sciences, Gregor-Mendel-Strasse 33, 1180 Vienna, Austria; 15 Department of Safety and Quality of Fruit and Vegetables, Max Rubner-Institut, Federal Research Institute of Nutrition and Food, Haid-und-Neu-Strasse 9, 76131 Karlsruhe, Germany; 16 Department of Toxicology, University of Würzburg, Versbacher Strasse 9, 97078 Würzburg, Germany; 17 Department of Food Chemistry and Toxicology, Faculty of Chemistry, University of Vienna, Währinger Strasse 38, 1090 Vienna, Austria; 18 Institute for Nutrition and Food Science, Rheinische Friedrich-Wilhelms-University Bonn, Fiedrich-Hirzebruch-Allee 7, 53115 Bonn, Germany; 19 Unit Reproductive and Genetic Toxicology, Federal Institute for Drugs and Medical Devices (BfArM), Kurt-Georg-Kiesinger Allee 3, 53175 Bonn, Germany; 20 Charité-Universitätsmedizin Berlin, Charitéplatz 1, 10117 Berlin, Germany; 21 Paul-Ehrlich-Institute, Paul-Ehrlich-Strasse 51-59, 63225 Langen, Germany; 22 Institute of Agricultural and Nutritional Sciences, Martin-Luther-University Halle-Wittenberg, Weinbergweg 22, 06120 Halle (Saale), Germany; 23 Department of Toxicology, Leibniz Research Centre for Working Environment and Human Factors (IfADo), Ardeystrasse 67, 44139 Dortmund, Germany

## Abstract

Since 2006, the responsible regulatory bodies have proposed five health-based guidance values (HBGV) for bisphenol A (BPA) that differ by a factor of 250,000. This range of HBGVs covers a considerable part of the range from highly toxic to relatively non-toxic substances. As such heterogeneity of regulatory opinions is a challenge not only for scientific risk assessment but also for all stakeholders, the Senate Commission on Food Safety (SKLM) of the German Research Foundation (DFG) analyzed the reasons for the current discrepancy and used this example to suggest improvements for the process of HBGV recommendations. A key aspect for deriving a HBGV is the selection of appropriate studies that allow the identification of a point of departure (PoD) for risk assessment. In the case of BPA, the HBGV derived in the 2023 EFSA assessment was based on a study that reported an increase of Th17 cells in mice with a benchmark dose lower bound (BMDL_40_) of 0.53 µg/kg bw/day. However, this study does not comply with several criteria that are important for scientific risk assessment: (1) the selected end-point, Th17 cell frequency in the spleen of mice, is insufficiently understood with respect to health outcomes. (2) It is unclear, by which mechanism BPA may cause an increase in Th17 cell frequency. (3) It is unknown, if an increase of Th17 cell frequency in rodents is comparably observed in humans. (4) Toxicokinetics were not addressed. (5) Neither the raw data nor the experimental protocols are available. A further particularly important criterion (6) is independent data confirmation which is not available in the present case. Previous studies using other readouts did not observe immune-related adverse effects such as inflammation, even at doses orders of magnitude higher than in the Th17 cell-based study. The SKLM not only provides here key criteria for the use of such studies, but also suggests that the use of such a “checklist” requires a careful and comprehensive scientific judgement of each item. It is concluded that the Th17 cell-based study data do not represent an adequate basis for risk assessment of BPA.

## Introduction

Bisphenol A (BPA) is a high production volume chemical widely used in the manufacture of polycarbonate plastics and epoxy resins, among other applications. Each year, approximately 10 million tons of BPA are manufactured worldwide. Due to its endocrine-disrupting properties, several restrictions on its production and use in consumer products have been put in place. However, the risk assessment of BPA has been debated for decades and remains controversial (Hengstler et al. 2011). The Senate Commission on Food Safety (SKLM) analyzed the reasons for discrepancies in the assessment of BPA and used this example to suggest improvements for the process of HBGV recommendations.

## Large discrepancies in the points of departure obtained from different studies

The responsible authorities have reported tolerable daily intake values (TDI) for BPA that differ by a factor of 250,000 (Table 1). This factor covers a considerable part of the toxicological classification of chemicals available worldwide—from the most toxic to the least toxic substances. Several circumstances have led to these extremely divergent assessments, with the difficulty in selecting scientifically adequate studies to derive a point of departure (PoD) being one of the most important. In 2006, the European Food Safety Authority (EFSA 2007) established a TDI of 50 µg/kg bw/day (Table 1), which was reaffirmed in 2008 and 2010 (EFSA 2008, 2010). This threshold value was based on a three-generation study in rats (Tyl et al. 2002) and a two-generation study in mice (Tyl et al. 2008a, b), in which BPA was found to reduce body weight and the weights of both, livers and kidneys. An overall no observed adverse effect level (NOAEL) of 5 mg BPA/kg bw/day was derived, and an uncertainty factor of 100 was applied. This TDI was accepted by most regulatory agencies worldwide (review: (Hengstler et al. 2011)). In 2015, EFSA derived a provisional TDI of 4 μg/kg bw/day based on toxic effects on the kidneys of mice in the two-generation reproductive toxicity study (Tyl et al. 2008a, b), taking into account remaining uncertainties for effects on the mammary gland, reproductive system, neurobehavioral system, immune system, and metabolism by applying an additional uncertainty factor (EFSA 2015).

**Table 1:** Tolerable daily intake (TDI) of bisphenol A since 2006 and the corresponding studies from which the points of departure were derived

Year	TDI	Point of departure	Source
2006	50 µg/kg bw/day	Decreased organ weight of rodents (Tyl et al. 2008a, b, 2002)	EFSA (2007, 2008)
2015	4 µg/kg bw/day (provisional)		EFSA (2015)
2021	0.04 ng/kg bw/day^a^	Increased levels of Th17 cells in spleens of mice exposed to 100 nM BPA in drinking water (Luo et al. 2016). BMDL_40_ calculated by EFSA: 0.53 µg/kg bw/day	EFSA (2021)
2023	0.2 ng/kg bw/day^b^		EFSA (2023)
2023	0.2 µg/kg bw/day	Reduced sperm count in adult rats BMDL_10_: 26 µg/kg bw/day (Liu et al. 2013); NOAEL: 50 µg/kg bw/day (Srivastava and Gupta 2018)	BfR (2023)
Exposure of the European population via food (based on data from 2008 to 2012)^c^: Adults: 0.1–0.4 µg/kg bw/day Children: 0.2–0.9 µg/kg bw/day			

^a^Suggested in the draft opinion published for public consultation (EFSA 2021)

^b^TDI of the final opinion

^c^Exposure assessment was performed by EFSA (EFSA 2015) and refers to the range between the minimum lower bound at the mean level of exposure and the maximum upper bound at the 95th percentile level of exposure

In its most recent assessment (EFSA 2023), EFSA used a mouse study in which pregnant dams were exposed to BPA via drinking water during and after pregnancy, followed by analyzing the Th17 cell (a type of T helper cells) frequency in the spleens of the offspring (Luo et al. 2016). The authors reported an increase in Th17 cells with only 100 nM of BPA in drinking water, equivalent to 4.75 µg/kg bw/day. Based on this, a BMDL_40_ of 0.53 µg/kg bw/day was used by EFSA to derive a TDI of 0.2 ng/kg bw/day, after applying an overall uncertainty factor of 50 (Table 1). In contrast, the German Federal Institute for Risk Assessment (BfR) based its derivation of a TDI on an end-point of the reproductive system (BfR 2023). Specifically, BfR selected two studies that reported reduced sperm counts in rats with a BMDL_10_ of 26 µg/kg bw/day and a NOAEL of 50 μg/kg bw/day, respectively (Liu et al. 2013; Srivastava and Gupta 2018), resulting in a TDI of 0.2 µg/kg bw/day. Considering the most recent TDI derived by EFSA 2023 (0.2 ng/kg bw/day), dietary exposure of an adult European population (approximately 0.1–0.4 µg/kg bw/day^1^) would exceed the TDI by a factor of 500–2000. For children (approximate exposure 0.2–0.9 µg/kg bw/day^1^), this factor would be up to 4500. These exposure estimates are mainly based on data from 2008 to 2012 and may not accurately reflect current dietary exposure (EFSA 2015; EFSA 2023), because exposure is expected to have decreased due to regulatory measures (BfR 2023).

The design of the Th17 cell-based study by Luo et al. used by EFSA as a basis for BPA risk assessment involved different steps, including exposure of pregnant dams to 10, 100, and 1000 nM BPA in drinking water (equivalent to 0.475, 4.75, and 47.5 µg/kg bw/day, respectively) from gestational day 0 to postnatal day 21, followed by analysis of the offspring mice on postnatal days 21 and 42. Splenocytes were isolated from the mouse spleen, suspended in culture medium, plated into culture dishes, stimulated with phorbol 12-myristate 13 acetate and monensin, stained with anti-CD4 and anti-IL-17 antibodies, and finally analyzed by fluorescence-activated cell sorting (FACS). This procedure was reported to result in an increase of the Th17 cell frequency from ~ 1.2% (controls) to ~ 2.1% in female mice with 100 nM BPA, which further increased to ~ 3.2% with 1000 nM BPA. Based on the observed effects on Th17, a NOAEL of 0.475 µg/kg bw/day and a LOAEL of 4.75 µg/kg bw/day were identified (BfR 2023; EFSA 2023; Luo et al. 2016).

## Critical discussion of the study by Luo and colleagues, and use of its data as a point of departure (PoD) for risk assessment

The choice of the end-point of the Th17 mouse study (Luo et al. 2016) as a PoD for risk assessment of BPA led to a critical discussion by several scientific bodies (*e.g.*, BfR 2022; BfR and EFSA 2023; EMA and EFSA 2023), mainly focusing on the following aspects:

As central point of criticism, the relationship between the reported increase in Th17 cells in the spleen of mice and adverse effects, such as, for example, tissue inflammation, is unclear. It is also unclear whether an increase in Th17 cells is relevant to humans. The SKLM agrees with this criticism, considering that other studies in which experimental animals were exposed to much higher doses than applied by Luo et al. (2016), such as the NTP CLARITY-BPA program (NTP 2018), did not observe evidence for inflammation or other immune-related adverse effects. It is well known that the proportion and activity of Th17 cells is influenced by several factors, including the gut microbiota or infections (Ang et al. 2020; Huber et al. 2012). Therefore, toxicological studies focusing on Th17 cells should take these potential confounding factors into account. An appropriate strategy would be to replicate the Th17 cell study with a design that includes also higher doses of BPA to elucidate if a further increase of the splenic Th17 cells can be induced and if this is associated with adverse effects, such as inflammation.

Another unclear aspect is the mechanism by which BPA may cause an increase in Th17 cell frequency. Although much is known about the mechanisms and receptors through which BPA may act, for example estrogen receptors ERα and ERβ, pregnane X receptor (PXR), the estrogen receptor-related receptor (ERR), and the thyroid hormone receptor (TR) (Hengstler et al. 2011), no attempt has been made to elucidate if these mechanisms are relevant for the reported phenotype with increased Th17 cells, although this could be achieved, for example, by studies in cells or mice in which the candidate mechanisms are deleted.

The relevance of the mouse model used in Luo et al. (2016) is difficult to assess. Even if the relationship of Th17 cells and some adverse effects would be known, the relevance and predictivity of the model concerning the human situation should be ascertained. Regulatory decisions should only be based on accepted scientific end-points that are considered relevant to humans (Cöllen et al. 2024; Pallocca and Leist 2022). There are some obvious differences between humans and mice in immune system regulation, in metabolism, and in the microbiome. The transfer between models and humans is easier, if studies provide a mechanistic rationale for why a compound causes certain effects and how these effects are related to adversity (Leist et al. 2017). This is particularly difficult for descriptive studies.

Moreover, toxicokinetics have not been addressed in the study of Luo et al. (2016). The SKLM suggests that at least the concentrations of BPA (and its metabolites) in the pups should be analyzed to see if they increase with higher concentrations of BPA in drinking water. Moreover, major differences in the toxicokinetics of BPA between rodents and humans are known (Collet et al. 2015; Hengstler et al. 2011). For example, BPA undergoes extensive enterohepatic recycling in rodents, in contrast to humans. To allow extrapolation to humans, toxicokinetic data of the animal model used are pivotal. This is even more critical when using complex animal models, such as the exposure of pregnant dams, where both, toxicokinetics in the mothers and placental transfer to the embryos are critical.

Additionally, the quality of the documentation of data and experimental procedures of Luo et al. (2016) is insufficient and does not meet international standards (DFG 2022). The original (raw) data and experimental protocols (*e.g.*, of the FACS analyses) are not available or incomplete. A critical weakness of the study is the used animal diet. In material and methods, it is written that a standard chow was given to the animals. However, the composition of this diet and the manufacturer were not indicated. It is important to figure out that rodent standard diets usually contain soy protein and thus substantial quantities of isoflavones (if not specified as “free of isoflavones”) exerting estrogenic effects. Therefore, they are unsuitable for studying effects of endocrine-disrupting substances, as they may influence the results. Moreover, effects of isoflavones on Th17 cells have been described (Kojima et al. 2015; Shu et al. 2024).

In conclusion, the study by Luo et al. (2016) refers to an intermediate parameter without a proven association with an adverse effect, without toxicokinetic data being collected and without raw data and protocols being documented. Such a pilot study may serve to generate hypotheses for follow-up work, *e.g.,* on a possible relevance of Th17 cells in the hypothesized BPA-mediated inflammatory effects. However, considering the discussed shortcomings and the fact that numerous published animal studies on BPA using doses several orders of magnitude higher than the BMDL reported by Luo et al. (2016) did not observe any BPA-associated tissue inflammation in histological investigations, this Th17 study should not serve as a basis for risk assessment.

Another critical aspect of ensuring high-quality risk assessment, which may have been neglected in the past, is the need for independent confirmation of data. Centuries of scientific theory and practice have shown that data that differ from previous canonical knowledge can only be considered valuable after they have undergone a test of reproducibility. For BPA, no dose-dependent immunological symptoms were observed in the research program CLARITY-BPA (NTP 2018), even though these studies used higher doses than those in Luo et al. (2016). The discrepancy between the Th17 study with its positive result at very low doses (Luo et al. 2016) and the negative findings of the research program CLARITY-BPA will remain an unsatisfactory situation until clarified experimentally. It is understandable that scientific bodies responsible for risk assessment find themselves in a difficult situation given the large number of studies with partially contradictory results. Thus, it would be an extremely important step forward, if in such—generally relatively rare—contradictory constellations, the responsible agencies would be authorized to commission a clarification study that is scientifically well-designed and sufficiently powered. Currently, most regulatory bodies in Europe usually evaluate published or submitted data, but they do not conduct or commission additional experiments themselves. However, if data for substances are contradictory or extremely diverging, it would be useful if this would become possible in the future.

## Criteria to identify adequate studies for risk assessment

To avoid problems in the future, such as those discussed above, criteria regarding the quality standards of studies that serve as a basis for risk assessment should be defined (Table 2). It is essential that the analyzed parameters can be related to an adverse outcome in the applied model and that a transferability of this to humans is plausible. If a study identifies an association between a test substance and an intermediate end-point whose relationship to an adverse outcome is unclear, this may indicate a research need, but should not serve as a basis for risk evaluation. If mechanistic relationships remain unclear, the transferability of model data to humans is difficult to judge. Numerous high-quality studies have already integrated toxicokinetics in the past. This should be mandatory for the experimental designs in future. The test compound should be quantified in the administered medium, such as drinking water and diet. It should also be known (and excluded) that additional exposure may occur, for example, due to background exposure (in this case to BPA) being potentially caused by the polycarbonate cages, contamination of the diet or other sources (any type of plastic material devices and tools being used in the respective setting). Potential effects of other feed ingredients or contaminants, such as isoflavones or aflatoxins, influencing certain end-points should be also considered or avoided. Moreover, the concentration–time curve in blood and, ideally, in target tissues of toxicity should be determined including analysis of potential metabolites. In general, the study design and reporting of data should be based on standardized, generally accepted criteria (*e.g.*, OECD or ARRIVE^2^ (Animal Research: Reporting of In Vivo Experiments). In principle, it should be self-evident that raw data and study protocols are made available, as comprehensive supplements to publications or at least upon request. Unfortunately, this is often not the case in current practice. The preferred option is that the material is part of a publication deposited in a data base, as modern academic labs have a volatile personnel situation, and data retrieval at later time points is often not possible. And last not least, independent data confirmation is critical, particularly when a study is not in line with the present state of knowledge.

**Table 2:** Suggested criteria for studies that serve as a basis for risk evaluation

(1) There should be a plausible connection, and some type of dose-concordance between the analyzed parameters and adverse effects
(2) The mechanism causing an adverse effect should be known
(3) The applied model (for example, a specific test in a laboratory animal species) should fulfill some minimum requirements concerning relevance and predictivity for the human situation (justified by robust historical experience or by some form of a readiness evaluation/validation)
(4) Toxicokinetics (including potential metabolites) should be adequately addressed
(5) Experimental quality requirements (as described, *e.g.*, in OECD Guidelines, the ARRIVE criteria or similar standards) should be fulfilled. In particular, the raw data and experimental protocols should be accessible
(6) Independent data confirmation is mandatory, particularly, when the results of an experiment are not in agreement with previous studies
(7) Additional exposure with the target compound from other sources (*e.g.*, plastic material) or exposure to other end-point critical compounds from feed (*e.g*., isoflavones) should be considered or avoided

It is important to consider that the criteria in Table 2 should not be used as a formal checklist leading to a not reflected exclusion of studies from the risk assessment process if one or even several of the criteria discussed here are not met. Rather, a comprehensive analysis and interpretation of the totality of evidence is required, which in some cases may be a complex challenge. Nevertheless, in the case of the study by Luo and co-workers, it appears clear that this work does not provide an adequate basis for the risk assessment process.

## Data Availability

Data sharing not applicable to this article as no datasets were generated or analysed during the current study.

## Footnotes

1 The overall given exposure encompasses the range between the minimum lower bound (LB) average exposure and the maximum upper bound (UB) at the 95th percentile exposure.

2 https://arriveguidelines.org/

## Declarations

### About the Senate Commission on Food Safety (SKLM)

The SKLM provides scientific advice on food safety issues to the DFG Senate as well as to federal/state governments and other authorities. In recent years, the SKLM has published multiple scientific articles and has issued several scientific opinions on topics such as acetaldehyde, fluoride, nitrate and nitrite in the context of food safety. Further information: https://www.dfg.de/en/dfg-profile/statutory-bodies/senate/food-safety
